# Conventional dose versus dose escalated radiotherapy including high-dose-rate brachytherapy boost for patients with Gleason score 9–10 clinical localized prostate cancer

**DOI:** 10.1038/s41598-021-04233-4

**Published:** 2022-01-07

**Authors:** Hideya Yamazaki, Gen Suzuki, Norihiro Aibe, Daisuke Shimizu, Takuya Kimoto, Koji Masui, Ken Yoshida, Satoaki Nakamura, Haruumi Okabe

**Affiliations:** 1 Department of Radiology, Graduate School of Medical Science, Kyoto Prefectural University of Medicine, 465 Kajiicho Kawaramachi Hirokoji, Kamigyo-ku, Kyoto, 602-8566 Japan; 2 Department of Radiology, Kansai Medical University, Hirakata, 573-1010 Japan; 3 Department of Radiology, Ujitakeda Hospital, Uji-City, Kyoto Japan

**Keywords:** Oncology, Urological cancer

## Abstract

As several recent researches focus on the importance of Gleason 9–10, we examine the role of radiotherapy dose escalation in those patients. We analyzed 476 patients with Gleason score 9–10 prostate cancer treated with radiotherapy. Of them, 127 patients were treated with conventional-dose external beam radiotherapy (Conv RT) and 349 patients were treated with high-dose radiotherapy (HDRT; 249 patients received high-dose-rate brachytherapy boost + external beam radiotherapy [HDR boost] and 100 patients received intensity-modulated radiotherapy [IMRT]). We compared these treatment groups using multi-institutional retrospective data. The patients had a median follow-up period of 66.3 months. HDRT showed superior biochemical disease-free survival (bDFS) rate (85.2%; HDR boost 84.7% and IMRT 86.6%) to Conv RT (71.1%, *p* < 0.0001) at 5 years, with a hazard ratio of 0.448. There were borderline difference in prostate cancer-specific mortality (PCSM; 4.3% and 2.75%, *p* = 0.0581), and distant metastasis-free survival (DMFS; 94.4% and 89.6%, *p* = 0.0916) rates at 5-years between Conv RT and HDRT group. Dose escalated radiotherapy showed better bDFS, borderline improvement in PCSM, and equivocal outcome in DMFS in with clinically localized Gleason 9–10 prostate cancer.

## Introduction

Recently, the concept of very high-risk factors was introduced into the risk classification of prostate cancer, and patients with these factors are considered to have the worst prognosis. Very high-risk factors include primary Gleason pattern 5 or more than four biopsy cores with a Gleason score of 8–10 or clinical stage T3b–T4^1,2^. Of these, Gleason pattern 5 is considered one of the most important factors for prognosis because it is associated with aggressive disease^2–4^. Kuban et al. reported the importance of a Gleason score of 9 or 10 as a predictive factor for prostate cancer-specific mortality (PCSM)^3^. Sabolch et al. also reported that the presence of Gleason pattern 5 in biopsy specimens is the strongest prognostic factor for all clinical outcomes, including PCSM and overall survival (OS), after external beam radiotherapy (EBRT) for T1–T4 prostate cancer^4^.

A comparative outcome analysis revealed that EBRT plus brachytherapy (BT) showed the best outcome in terms of PCSM and longer time to distant metastasis (distant metastasis-free survival [DMFS]) than EBRT and radical prostatectomy^5^, which prompted us to examine the role of dose escalation (including BT boost) to confirm these effects. To explore these findings in a large cohort, we used freely available public data on EBRT, high-dose rate (HDR) BT boost^6^, and intensity-modulated radiotherapy (IMRT) performed at our institutions^7^. Therefore, the aim of the present study was to investigate the role of dose escalation (including HDR boost) in radiotherapy in patients with clinically localized Gleason score 9–10 prostate cancer.

## Methods

### Patients

We retrospectively analyzed the data of patients treated with EBRT + BT (249 patients who received high-dose BT boost, from an open data source for public use)^6^ and EBRT (127 patients who received conventional-dose EBRT [Conv RT; from open data] and 100 patients who received high-dose RT [HDRT] with IMRT [from open data] performed at Uji-Takeda Hospital])^7^ (Table 1, Supplemental Table 1). The patient eligibility criteria were as follows: treatment with EBRT + BT or EBRT alone, clinical tumor–node–metastasis stage T1–T4, N0M0 with Gleason score 9–10, histology-proven adenocarcinoma, and availability and accessibility of pretreatment data (initial prostate-specific antigen [PSA] level, Gleason score sum, and T classification). We defined PSA failure according to the Phoenix definition (nadir + 2 ng/mL). The Common Terminology Criteria for Adverse Events version 4.0 was used for the toxicity analysis. Toxic effects occurring within 90 days after radiotherapy completion were considered acute, and toxic effects occurring after that 90-day period were considered late. All patients in the study by the Uji-Takeda group provided written informed consent, and patients in the public data source provided informed consent during the process of building public data. This study was conducted in accordance with the Declaration of Helsinki and received institutional review board approval (Kyoto Prefectural University of Medicine Institute, approval no. ERB-C-1403).

**Table 1:** Comparison of backgroud patients characteristics between Conv RT and DeRT group.

Variables	Strata	Conv group		HDRT group		*p*-value
		Conv EBRT (n = 127)		IMRT + HDR boost (n = 349)		
		No. or median [range]	(%)	No. or median [range]	(%)	
Age		71 [60, 89]		71 [60, 86]		0.487
T category	2	33	(26%)	164	(47%)	** < 0.0001**
	3a	49	(39%)	130	(37%)	
	3b	35	(28%)	50	(14%)	
	4	10	(8%)	5	(1%)	
iPSA		31.54 [5.32, 352]		16.00 [3.09, 500]		** < 0.0001**
Gleason score	9	123	(97%)	313	(90%)	**0.014**
	10	4	(3%)	36	(10%)	
Prescribed dose (BED)	(Gy)	168 [163, 168]		244.67 [172, 303]		** < 0.0001**
Hormonal therapy follow-up	Yes	127	(100%)	335	(96%)	0.026
	Duration (Months)	10.00 [4.00, 140]		38.00 [3.00, 128]		** < 0.0001**
	No	0	(0%)	14	(4%)	
	(Months)	83.5 [11.2, 145]		61.0 [2.00, 158]		** < 0.0001**

Characteristics and treatment factors of patients.

*HDR boost* high dose rate brachytherapy boost, *EBRT* external beam radiotherapy.

EQD 2 Gy = n × d × (α/β + d)/(α/β + 2) (α/β = 1.5 Gy, n = fraction number, d = single dose).

BED = n × d × (1 + d/(α/β)); (α/β = 1.5 Gy, n = fraction number, d = single dose).

Bold values indicate statistically significance between Conv group and DeRT group.

### Treatment planning

#### HDR BT boost

Multi-institution data were obtained from an open data source^6^, and the detailed method of applicator implantation has been described elsewhere^8^. Table 2 shows the detailed schedules of the combination of HDR boost and EBRT. HDR boost used 31.5 Gy (10.5–31.5 Gy) and EBRT used 30 Gy (30–51 Gy) as the median dose. The median fraction size of HDR boost was 6.3 Gy (5–11 Gy), and that of EBRT was 3 Gy (2–3 Gy).

**Table 2:** Detailed schedule of radiotehrapy and BED or EQD2 for each treatment.

Prescribed dose	PT no.	(%)	BED	EQD 2 Gy
**Conv. RT (n = 127)**				
70 Gy/35fr	13	(15%)	163	70
72 Gy/36fr	114	(133%)	168	72
**IMRT (n = 100)**				
74 Gy/36fr	32	(82%)	172	74
78 Gy/39fr	16	(41%)	182	78
74.8 Gy/34fr	24	(62%)	184	79
80 Gy/40fr	28	(72%)	186	80
**HDR boost (n = 249)**				
20 Gy/2fr + EBRT30Gy/15fr	1	(0.4%)	223	95
10.5 Gy/1fr + EBRT 51 Gy/17fr	1	(0.4%)	237	101
18 Gy/2 fr + EBRT 39 Gy/13 fr	36	(14%)	243	104
11 Gy/1fr + EBRT51Gy/17fr	41	(16%)	244	104
31.5 Gy/5fr + EBRT 30 Gy/10fr	132	(53%)	253	109
20 Gy/2fr + + EBRT × 46 Gy/23fr	2	(1%)	260	111
25 Gy/5fr + EBRT 51 Gy/17fr	1	(0.4%)	261	112
21 Gy/3fr + EBRT 51 Gy/17 fr	2	(1%)	272	116
18 Gy/2 fr + EBRT51 Gy/17fr	31	(12%)	279	119
21 Gy/2 fr + EBRT 45 Gy/15fr	2	(1%)	303	130

EQD 2 Gy = n × d × (α/β + d)/(α/β + 2) (α/β = 1.5 Gy, n = fraction number, d = single dose).

BED = n × d × (1 + d/(α/β)); (α/β = 1.5 Gy, n = fraction number, d = single dose).

#### EBRT

Table 2 shows the detailed schedule of Conv RT and HDRT group, including conventional two-dimensional treatment planning, three-dimensional conformal radiotherapy planning, and IMRT planning. Some EBRT data were obtained from a freely accessible dataset (n = 155)^6^, and 72 image-guided IMRT procedures using helical tomotherapy were performed at the Department of Radiology of Uji Takeda Hospital. The technique of image-guided IMRT using helical tomotherapy has been described elsewhere^7^. We prescribed a dose at D95 (95% of the planning target volume received at the least prescribed dose) of 74.8 Gy/34 fractions (2.2 Gy/fraction, n = 62) from June 2007 to May 2009 and modified the prescribed dose by reducing to 74 Gy/37 fractions (2 Gy/fraction, n = 79) from June 2009 to September 2013^7^ at Uji Takeda Hospital. Eighty-seven patients (12 in HDRT and 75 in Conv RT group) received pelvic node prophylactic irradiation.

### Statistical analysis

StatView 5.0 and the EZR statistical package were used for statistical analyses^9^. The EZR statistical package was used for competing risk analysis (Gray analysis and Fine–Gray model). Percentages were analyzed using chi-square tests, and Student’s *t*-test was used for normally distributed data. The Mann–Whitney U-test and Kruskal–Wallis test for skewed data were used to compare means or medians. The Kaplan–Meier method was used to analyze the biochemical disease-free survival (bDFS), DMFS, and OS rates. Gray analysis was used for assessing PCSM. Comparisons were made using log-rank tests or Gray analysis. A cause-specific analysis (death of other causes was assigned as a censor variable) was applied to the bDFS, OS, and DMFS rates, whereas competing risk analysis was used for the PCSM rate. Cox proportional hazard models for bDFS, DMFS, and OS and the Fine-Gray model for PCSM were used for univariate and multivariate analyses. Statistical significance was set at *p* < 0.05.

## Results

### Patient and tumor characteristics

A total of 476 patients with Gleason 9–10 (very high-risk) clinically localized prostate cancer were treated with HDR boost (n = 249) or EBRT (n = 227). The median patient age was 71 years (range 60–89 years). The clinical characteristics of the patients are presented in Table 1. The median follow-up duration of the entire cohort was 66.3 months (range 2–158 months), with a minimum of 1 year for surviving patients or until death.

Table 1 compares the background patient characteristics between the Conv RT and HDRT groups. Supplemental Table 2 shows the patient characteristics among the Conv RT, HDR boost, and IMRT groups.

### Biochemical control rate (bDFS)

**Figure 1:**
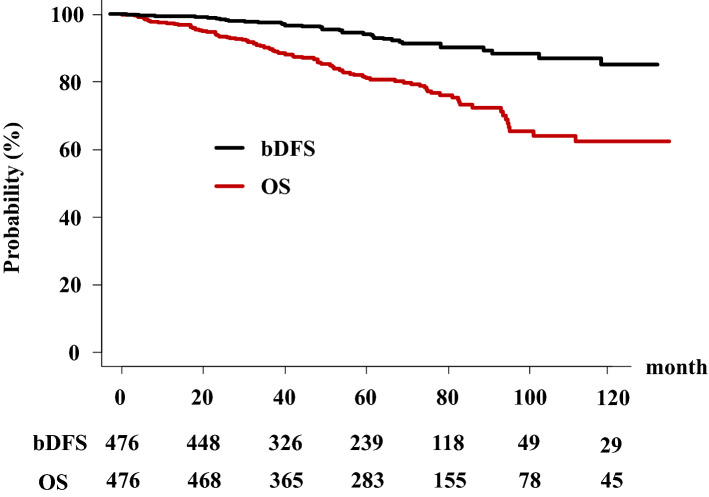
Biochemical disease-free survival (bDFS) and overall survival rate (OS) in patients with clinical localized Gleason 9–10 prostate cancer.

**Figure 2:**
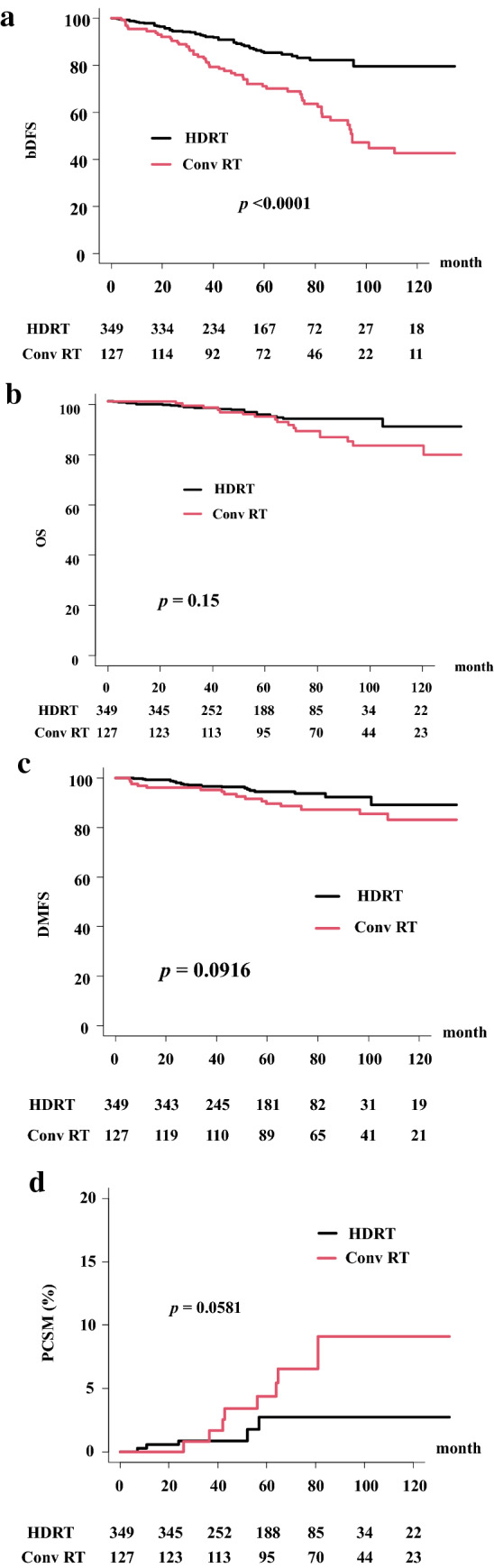
Comparison between Conv RT and HDRT groups. (**a**) Biochemical disease-free survival (bDFS) between Conv RT and HDRT. (**b**) Overall survival rate (OS) between Conv RT and HDRT. (**c**) Distant metastasis free survival rate (DMFS) between Conv RT and HDRT. (**d**) Prostate cancer specific mortality (PCSM) between Conv RT and HDRT.

**Figure 3:**
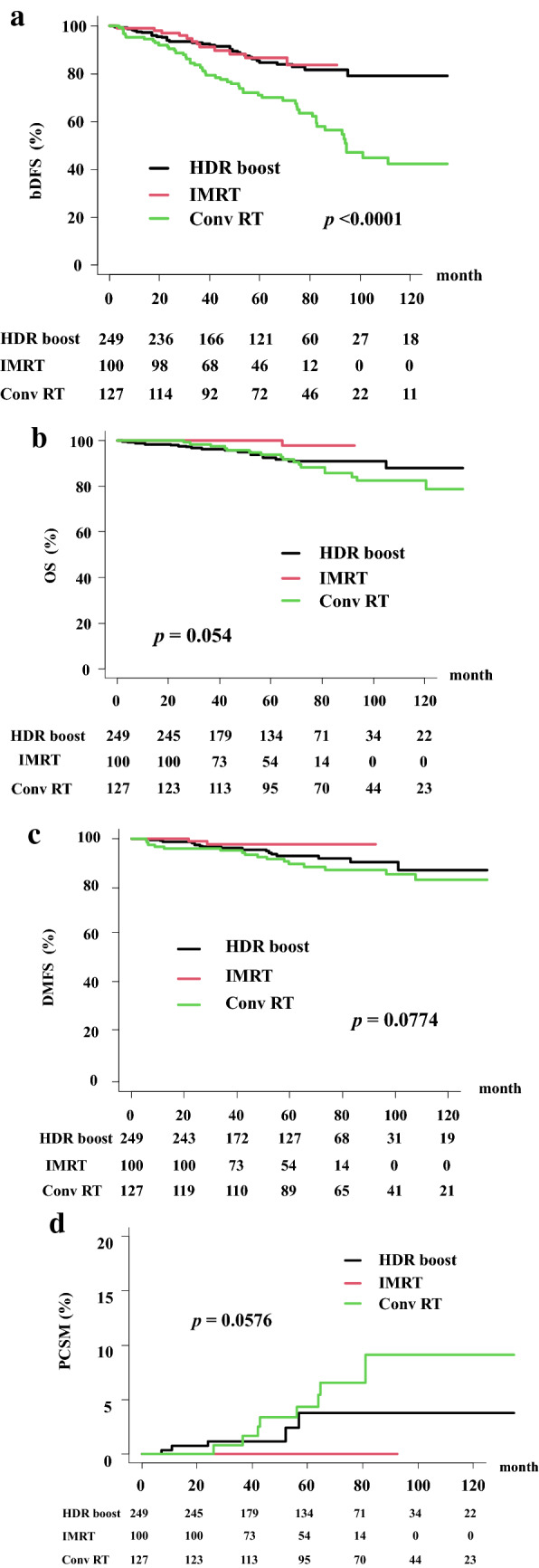
Comparison among three groups. (**a**) Biochemical disease-free survival (bDFS) among Conv RT, HDR boost and HD IMRT. (**b**) Overall survival rate (OS) among Conv RT, HDR boost and HD IMRT. (**c**) Distant metastasis free survival rate (DMFS) among Conv RT, HDR boost and HD IMRT. (**d**) Prostate cancer specific mortality (PCSM) among Conv RT, HDR boost and HD IMRT.

In the total population, the actuarial 5-year bDFS rate was 81.1% (95% confidence interval [CI] 76.7–84.7%) (Fig. 1). The HDRT group showed a higher bDFS rate (85.2%, 95% CI 80.2–89.0%) than the Conv RT group (71.1%, 62.0–78.5%, *p* < 0.0001) at 5 years (Fig. 2). In detail, there is no difference between the BT group (bDFS rate of 84.7%, 78.6–89.2%) and the high-dose IMRT group (86.6%, 76.8–92.4%) at 5 years and both of them showed superiority to Conv RT group (Fig. 3).

As shown in Table 3, the predictors of biochemical control in univariate analysis included the treatment group, T classification, and baseline PSA level. In multivariate Cox regression analysis, the HDRT group showed superior outcomes to those of the Conc RT group (hazard ratio 0.448, 95% CI 0.283–0.7081, *p* = 0.0006) and advanced T classification 3 ≤ showed statistically significant influence. Among three groups, both HDR boost (HR 0.382) and IMRT (HR 0.375) showed statistically significant improvement than conv RT in univariate analysis (Table 2).

**Table 3:** Uni- and multi-variate analysis for biochemical control rate using Cox proportional hazards model.

Variable	Strata	Univariate analysis			Multivariate analysis		
		HR	95% CI	*p*	HR	95% CI	*p*
Age, years	≤ 70	1	(Referent)	–	1	(Referent)	–
	71 ≤	1.258	0.843–1.876	0.2613	1.283	0.856–1.923	0.2278
T classification	≤ 2	1	(Referent)	–	1	(Referent)	–
	3 ≤	3.022	1.849–4.940	** < 0.0001**	0.401	0.238–0.675	**0.0006**
Gleason score	9	1	(Referent)	–	1	(Referent)	–
	10	1.253	0.581	0.5658	0.938	0.427–2.062	0.874
Pretreatment PSA (ng/mL)	≤ 20	1	(Referent)	–	1	(Referent)	–
	20 <	1.789	1.196–2.678	**0.0047**	1.077	0.695–1.669	0.7392
ADT duration (months)	≤ 33	1	(Referent)	–	1	(Referent)	–
	34 ≤	0.672	0.440–1.026	0.0658	0.966	0.604–1.545	0.8842
Treatment group	Conv. RT	1	(Referent)	–	1	(Referent)	–
	HDRT	0.38	0.255–0.567	** < 0.0001**	0.448	0.283–0.708	**0.0006**
	HDR boost	0.382	0.247–0.589	** < 0.0001**			
	IMRT	0.375	0.199–0.708	0.0025			

*CI* confidence interval, *HR* hazard ratio, *NA* not available, *HDR boost* high dose rate brachytherapy boost, *EBRT* external beam radiotherpay, *Conv RT* conventional radiotherapy.

Bold values indicate statistically significance.

### OS and DMFS rates

The OS rate was 94.3% (95% CI 91.5–96.3%) at 5 years and 86.8% (81.0–90.9%) at 10 years in the total population (Fig. 1). The OS rates in the HDRT group were 94.6% (91.1–96.8%) and 89.9% (80.8–94.8%) and those in the Conv RT group were 93.9% (87.6–97.0%) and 82.4% (72.5–89.0%) at 5 and 10 years, respectively (*p* = 0.15) (Fig. 2a). The HDR boost group showed OS rates of 92.5% (87.7–95.5%) at 5 years and 88.0% (78.8–93.4%) at 10 years, whereas the high-dose IMRT group showed OS rates of 100% at 5 years and 97.73% (84.94–99.68) at 10 years (Fig. 3b, p = 0.054).

The DMFS rate was 92.9% (95% CI 89.8–95.1%) at 5 years and 87.0% (80.6–91.4%) at 10 years in the total population (Fig. 2c). The HDRT group showed DMFS rates of 94.4% (90.8–96.6%) at 5 years and 89.1% (79.1–94.4%) at 10 years, whereas the Conv RT group showed DMFS rates of 89.6% (82.4–94.0%) at 5 years and 83.2% (72.9–89.8%) at 10 years (*p* = 0.0916). The HDR boost group showed DMFS rates of 92.9% (88.2–95.8%) at 5 years and 87.3% (77.1–93.1%) at 10 years, whereas the high-dose IMRT group showed a DMFS rate of 97.9% (91.7–99.5%) at both 5 and 10 years (Fig. 3c, p = 0.0774).

### PCSM

The cumulative PCSM rate was 1.14% (95% CI 0.6–1.9%) at 5 years and 3.12% (1.9–4.8%) at 10 years in the total population. The Conv RT group showed PCSM rates of 4.3% (1.6–9.3%) at 5 years and 9.1% (4.4–15.9%) at 10 years (*p* = 0.0581, Fig. 2d), with statistically borderline significance. For the HDRT group, the PCSM rate was 2.75% (1.2–5.4%) at both 5 and 10 years. The PCSM rate in the HDR boost group was 3.81% (1.6–7.4%) at 5 and 10 years and that in the IMRT group was 0% in 5 years (*p* = 0.0576, Fig. 3d).

### Late toxicity

Table 4 shows comparison of late toxicity between Conv and HDRT group. Equivocal gastrointestinal toxicity and higher genitourinary toxicity were found in HDRT group. In detailed analysis (Supplemental Table 2), HDR boost showed highest genitourinary toxicity and lowest gastrointestinal toxicity.

**Table 4:** Comparison between ConvRT and HDRT group for late toxicity.

	Grade	Conv group (n = 127)		HDRT Group (IMRT + HDR boost)(n = 349)		*p*-value
		No.	(%)	No.	(%)	
Gastrointestinal toxicity	0	101	(79.5%)	296	(84.8%)	0.59
	1	21	(16.5%)	296	(84.8%)	
	2	4	(3.1%)	42	(12.0%)	
	3	1	(0.8%)	9	(2.6%)	
Genitourinary toxicity	0	117	(92.1%)	0	(0.0%)	** < 0.001**
	1	5	(3.9%)	197	(56.4%)	
	2	4	(3.1%)	115	(33.0%)	
	3	1	(0.8%)	29	(8.3%)	

Significant value is given in bold.

## Discussion

Donald Gleason proposed a grading system for prostate cancer half a century ago, and the Gleason scoring system still has diagnostic importance or may even have a more central role at present^10^. Gleason identified five histological patterns (from the most well differentiated [Gleason pattern 1] to the least differentiated [Gleason pattern 5]), and this system, when combined with stage, has been shown to be prognostic for OS^10^. Many trials have confirmed the importance of this grading system, and several recent studies have focused on the importance of Gleason score 9–10^1–5^.

It is already well established that dose escalation improves bDFS. Many randomized controlled trials and meta-analysis studies have demonstrated the superiority of treatment with increased prescribed dose for localized prostate cancer^11–14^. Pollack et al. confirmed the superiority of the 78-Gy dose to the 70-Gy dose (the bDFS rates for the 70- and 78-Gy arms at 6 years were 64% and 70%, respectively)^13^. According to these notions, the Comprehensive Cancer Network Clinical Practice Guidelines in Oncology (2019) stated that a dose of 70 Gy in conventional fractions is not appropriate for patients with localized prostate cancer^1^. Therefore, we compared the outcomes of Conv RT using a prescribed dose of 70–72 Gy with higher-dose EBRT with IMRT with a prescribed dose of ≥ 74 Gy and HDR boost. In this study, we presented evidence that dose escalation, including HDR boost and IMRT, improves the biochemical control rate even in Gleason 9–10 prostate cancer based on a population of > 400 patients, which may be in line with the results of previous studies for the entire high-risk group^11–14^. Our findings may be beneficial for counseling individual patients with Gleason score 9–10 prostate cancer with respect to their treatment and prognosis.

BT has several merits that enable the delivery of higher doses of radiation to the target lesion while avoiding unnecessary higher irradiation to adjacent organs at risk, and is therefore considered one of the best radiotherapy techniques^15^. Additionally, the low α/β ratio of prostate adenocarcinoma cells implies higher sensitivity to large radiation doses per fraction than most other malignancies^15,16^. Therefore, better outcomes could be expected with dose escalation using hypofractionated schedules with HDR boost^16^. A few prospective studies and several retrospective studies have reported the merits of HDR boost^1,15–17^. These trials focused on low- and intermediate-risk prostate cancer. Therefore, little prospective data have been accumulated in high-risk groups, especially in patients with very high-risk prostate cancer, such as those with Gleason score 9–10. Thus, our study could provide useful information for making daily clinical decisions for very high-risk patients. We also investigated HDR monotherapy and reported a 5-year bDFS rate of 91.5% in Gleason 9–10 disease (n = 48)^18^, indicating that HDR monotherapy is also a promising procedure with good outcomes compared with Conv RT.

Some authors reported the superiority of HDR boost not only to Conv RT but also to high-dose EBRT (e.g., IMRT) in terms of the bDFS rate^19,20^. Spratt et al. reported the superior bDFS outcome of HDR boost in patients with intermediate-risk prostate cancer compared with high-dose IMRT alone (even at a dose of 86.4 Gy), but not in the high-risk group^20^. Furthermore, several studies observed improvement not only in terms of bDFS but also PCSM with dose escalation using BT^20–22^. Kishan et al. reported that EBRT + BT was associated with significantly lower PCSM rate (3%) than either radical prostatectomy (12%) or EBRT (13%) in Gleason 9–10 disease even after propensity adjustment^5^. In contrast, our data indicated that BT boost and IMRT did not translate into improved PCSM (HDR boost 3.81%, IMRT 0%). Muralidhar et al. also reported equivocal results between BT boost and radical prostatectomy in the Surveillance, Epidemiology and End Results cohort, with no difference in the 5-year PCSM (radical prostatectomy 6.0% vs. BT boost 5.7%)^23^. Although our shorter follow-up period did not allow concluding that HDRT could improve PCSM better than Conv RT, high-dose IMRT showed equivocal or superior outcome to HDR boost, which does not concur with previous data^5,20–22^.

BT has been facing a slow but progressive decline over the past decades. To overcome this problem, specific strategic interventions must be carried out in the field of national guidelines, education, research, and communication with patients and colleagues of other specialties in an interdisciplinary setting^24^.

For toxicity analysis, higher dose did not always elevate toxicity in gastrointestinal tract. IMRT and HDR boost could avoid higher dose to gastrointestinal organ, resulting in non-inferior toxicity profile to Conv RT group. Hydrogel spacer also could reduce GI toxicity not only in fresh case but also for reirradiation even though with ulcerative colitis^25^. However, higher dose was inevitably irradiated to genitourinary organs including urethra which is located inside of prostate, therefore higher toxicity was found in HDRT group especially in HDR boost group.

Androgen deprivation therapy (ADT) plays an important role in the treatment of high-risk prostate cancer. Zapatero et al. showed an improvement in 5-year bDFS with an additional 2-year adjuvant ADT from 81 to 90% after 6–82 Gy of EBRT in high-risk patients treated with three-dimensional conformal radiotherapy as neoadjuvant therapy^26^. We used long-term ADT, which could be one of the reasons for our good outcomes compared with previous studies. Furthermore, the good efficiency of ADT has been demonstrated in Japanese men, which can be attributed to the Japanese-specific high sensitivity to hormonal therapy^27^. However, a recent meta-analysis of trials of RT and ADT suggested that patients with Gleason score 9–10 prostate cancer had the greatest benefit from lifelong ADT, whereas the optimal treatment for those with Gleason score 8 prostate cancer might be long-term (but not lifelong) ADT^28^. We also observed that longer-term ADT use by > 2 years increases the occurrence of other causes of mortality in patients aged > 75 years^29^. Meticulous patient selection should be considered to maximize the efficacy of ADT without toxicity.

The present study had several limitations. The retrospective nature of the study confers limitations related to follow-up time. Moreover, the small sample size cannot reflect the entire population of patients with prostate cancer, which may limit the application of our findings.

## Conclusions

This study shows that dose-escalated radiotherapy results in improved bDFS, borderline improvement in PCSM, and equivocal outcomes in terms of DMFS in patients with Gleason 9–10 prostate cancer.

## Supplementary Information

Supplementary Tables.

## Data Availability

The data of HDR-BT and part of EBRT for this manuscript can be obtained from the public data base^6^ and other EBRT was can be obtained from the author upon reasonable request.
